# Vaccine Preparedness for the Next Influenza Pandemic: A Regulatory Perspective

**DOI:** 10.3390/vaccines10122136

**Published:** 2022-12-13

**Authors:** Norman W. Baylor, Jesse L. Goodman

**Affiliations:** 1 Biologics Consulting, Inc., Alexandria, VA 22314, USA; 2 Georgetown University Medical Center, Washington, DC 20057, USA

**Keywords:** regulatory, pandemic influenza, vaccines, SARS-CoV-2

## Abstract

The response to SARS-CoV-2 demonstrated the tremendous potential of investments in vaccine research and development to impact a global pandemic, resulting in the rapid development and deployment of lifesaving vaccines. However, this unprecedented speed was insufficient to either effectively combat initial waves of the pandemic or adapt in real time to new variants. This review focuses on opportunities from a public health oriented regulatory perspective for enhancing research, development, evaluation, production, and monitoring of safety and effectiveness to facilitate more rapid availability of pandemic influenza vaccines. We briefly review regulatory pathways and processes relevant to pandemic influenza, including how they can be strengthened and globally coordinated. We then focus on what we believe are critical opportunities to provide better approaches, tools, and methods to accelerate and improve vaccine development and evaluation and thus greatly enhance pandemic preparedness. In particular, for the improved vaccines needed to respond to a future influenza pandemic better and more rapidly, moving as much of the development and evaluation process as possible into the pre-pandemic period is critical, including through approval and use of analogous seasonal influenza vaccines with defined immune correlates of protection.

## 1. Introduction

The response to the SARS-CoV-2 global pandemic has demonstrated the tremendous potential of investments in vaccine research and development, resulting in the rapid development and deployment of vaccines that have saved millions of lives. However, even this unprecedented speed was insufficient to effectively combat initial waves of the pandemic or adapt in real time to new variants, leading to calls to further accelerate vaccine development for future outbreaks [1]. As the response to SARS-CoV-2 continues, there is a unique opportunity to learn from the successes and shortcomings of the current effort, as well as other recent outbreaks, to better prepare for and respond to future events, including the inevitable next influenza pandemic. 

In addition to the obvious challenges of speed, access, and trust, major interrelated issues common to both COVID-19 and pandemic influenza include the inability to predict and assess the transmissibility and severity of strains or variants with pandemic potential, lack of experience with new and emerging technologies, and broad gaps in human and financial resources between high and lower/middle income countries (LMIC) resulting in disparities in manufacturing capacity and regulatory oversight [2]. 

Pandemic influenza vaccine development presents unique advantages and challenges. These include a long history of production of approved, safe seasonal influenza vaccines, most of which are evaluated based on known surrogates of protection. However, current influenza vaccines are of suboptimal efficacy and often produced with obsolete manufacturing methods that are too slow to scale up to meet the needs of a rapidly spreading pandemic. For development of novel pandemic influenza vaccines, there is a unique opportunity to de-risk future pandemic use by testing efficacy and safety in the prevention of seasonal influenza during the pre-pandemic period.

Herein, we focus on opportunities, based on our regulatory and public health experience, for enhancements to facilitate more rapid availability of pandemic influenza vaccines. Addressing these opportunities is also expected to benefit pandemic and outbreak preparedness more generally, including for a wide variety of pathogens. We expect that strong, highly interactive, and science-based regulation by independent regulatory agencies can greatly contribute. We first briefly review relevant regulatory pathways and processes, including how they can be strengthened and globally coordinated. We then discuss opportunities to advance regulatory science [3] and provide better scientific approaches, tools, and methods to accelerate and improve vaccine development, evaluation and production and thus greatly enhance pandemic preparedness. 

## 2. Overarching Lessons from Developing Vaccines against Emerging Infectious Diseases

Perhaps the most important lesson from the COVID-19 pandemic and other outbreaks is the power of proactive engagement across sectors and agencies when objectives are clear and leadership, incentives, and resources are aligned to achieve public health goals. On the regulatory front, this includes interactive engagement between regulatory agencies and industry, timely development of regulatory guidance, and the use of regulatory flexibility and alternate pathways to speed access to approved or, where needed in emergency settings, investigational products.

In 2009 the world was faced with an H1N1 influenza pandemic with initial concern for potential high severity. Unlike outbreaks such as COVID-19, Zika, and Ebola, there were existing approved vaccines for seasonal influenza. However, it was unknown whether a standard dose of hemagglutinin (HA) antigen would suffice to stimulate a protective response. It was widely assumed, based on avian influenza threats such as H5N1, that two doses and addition of then novel adjuvants might be required. To accelerate the 2009 H1N1 vaccine response, the FDA, updating prior guidance [4], made clear that it would view a pandemic vaccine made with identical methods and facilities used for seasonal influenza as a strain-change modification of a licensed vaccine rather than as a new vaccine, thus not requiring large clinical studies. The use of the strain-change pathway accelerated and simplified vaccine development. However, development and manufacturing were nonetheless too slow to impact the first pandemic wave or begin to meet global needs. In addition, as novel adjuvants were not required, their regulatory status for potential usefulness in future pandemic vaccines remained unclear, with safety questions raised by the association of one non-US licensed adjuvanted vaccine with narcolepsy [5].

The challenges of the 2009 pandemic prompted the US government’s 2010 Public Health and Emergency Medical Countermeasures Enterprise (PHEMCE) review [6]. Major recommendations included advancing regulatory science to reduce risks and enhance speed in countermeasure development and providing multi-year sustainable support for development of priority countermeasures, including an emphasis on platform technologies and surge capacity utilizing flexible, threat-agnostic capabilities. These recommendations helped lead to investments in platform technologies, including mRNA, which were instrumental in the early COVID-19 response. However, as seen after the 2009 influenza pandemic and now with COVID-19, once a threat wanes so too does governmental attention. The resulting failure to make long-term investments required to support late-stage clinical development and manufacturing capacity for vaccines and other countermeasures without clear commercial prospects, such as pandemic influenza, impairs preparedness and disincentivizes industry engagement. 

The COVID-19 vaccine effort has illustrated both the power and shortcomings of efforts by governmental and non-governmental organizations (NGOs) to engage and incentivize needed actions by industry. Most notable has been the multi-billion-dollar US investment through Operation Warp Speed (OWS) funding “at-risk” development and production of vaccines by several manufacturers. The FDA helped further de-risk vaccine development by issuing guidance on SARS-CoV-2 vaccine licensure and Emergency Use Authorization (EUA) requirements early on and providing highly interactive advice and review to OWS-funded developers [7,8,9]. The NIH utilized its existing vaccine clinical evaluation network to facilitate studies of COVID-19 vaccine candidates funded by OWS. While OWS funding helped in promoting harmonization of clinical trial design, and sharing of blood samples for centralized testing, it left most specifics of clinical development, as well as ongoing public communication and interpretation of study results, at the discretion of the sponsors. This contributed to missed opportunities to optimize clinical trial outputs (for example comparability of results across differing vaccine products), as well as often confused public communication and messaging (for example, sponsors recommending boosters prior to regulatory review or decision-making by government officials). Furthermore, while beyond the scope of this article, the US effort did not include provisions to enhance global information sharing or vaccine access, even from developers it supported. The Coalition on Epidemic Preparedness Innovation (CEPI), an NGO formed to support collaboration around investments in vaccine preparedness for outbreaks, including prioritization of global access, funded several vaccine developers starting as early as January 2021 and partnered with WHO and others in the COVAX initiative to purchase and distribute vaccines for LMIC nations. However, absent sufficient initial funding, COVAX could not compete with others to purchase early vaccine, and global access was slow to ramp up. 

While beyond the scope of this article, there is an overarching need for a clear strategy for pandemic influenza research and development that goes beyond a list of desired studies to include planning and resources that cut across and integrate vaccines, therapeutics, diagnostics, public health, and medical care. This strategy requires close coordination of regulatory preparedness with pandemic preparedness. It should include collaborative development of a prioritized research agenda that engages relevant US agencies with distinct areas of expertise and equities (e.g., NIH, CDC, FDA, DOD, VA, CMS) in planning, governance, and implementation. Further, the effort should include close coordination with global partners such as WHO. There are precedents previously supported through the PHEMCE [6]. To succeed, such an enterprise must be empowered at high levels of government and be highly interactive with academic and industrial innovators and manufacturers. Early engagement of regulators is critical to ensure needed data are generated to support sound decision-making and public trust. With the above issues in mind, we now turn to a discussion of opportunities to speed and improve pandemic influenza vaccine regulation and development from early to late stages.

## 3. Regulatory Policy and Pathways

To ensure a rapid global pandemic response, while protecting people from unsafe or ineffective vaccines and preserving public confidence, NRAs must be able to evaluate vaccines rapidly and reliably for approval or, when needed, for access under emergency provisions (Table 1). This capability requires both science-based regulatory pathways and technical capacity. While we herein predominantly provide a US regulatory perspective, the needs are global. To accelerate vaccine access and avoid the potential for confusion when different nations apply different standards and guidance, preparing for the next pandemic requires global convergence of relevant regulatory requirements and pathways. 

There are four major expedited US FDA regulatory pathways to facilitate development and licensure of products to address unmet medical needs for serious conditions: fast track, breakthrough therapy, priority review and accelerated approval [10]. Designation of a vaccine under these mechanisms does not lower either the standards or the quality of data required for licensure. 

In a public health emergency approved products may not initially be available. FDA’s EUA, while not an approval pathway, is a mechanism to authorize use of unapproved medical products for a declared (or potential) public health emergency where no adequate, approved alternatives are available. The regulatory standard for an EUA is lower than that for approval, which requires clear-cut demonstration of safety and efficacy based on adequately controlled clinical trial(s). In contrast, an EUA can be granted if the FDA’s assessment indicates the product may be beneficial and that known and potential benefits are likely to outweigh known and potential risks [11]. As seen during the COVID-19 pandemic, the FDA may apply risk-based criteria to EUA, for example, requiring less supporting evidence for drug products intended to treat seriously ill patients than for vaccines given to millions of healthy individuals. 

Unlike use of an unapproved product under an investigational new drug application (IND), use under an EUA does not require informed consent, although recipients must be informed that the product is unapproved and provided information on its risks and benefits as well as potential alternatives. For products where sufficient evidence may not be initially available to support an EUA, there are potential mechanisms to allow for wide access under an IND, including so-called compassionate use or expanded access programs and treatment INDs. These mechanisms require informed consent and can be useful in circumstances where data are also being collected through ongoing controlled clinical trials, but access outside those trials may be desirable. 

As in the US, NRAs throughout the world are central to facilitating access to and maintaining trust in vaccines as they are responsible for independent oversight of their safety, quality, and efficacy. Yet, many do not have adequate capacity to provide such oversight, particularly in emergency situations [12]. The WHO has been pivotal in building global regulatory capacity through its prequalification program, which depends on and allows reference to evaluations of products by NRAs it deems “functional” [13], and its Emergency Use Listing Procedure which provides an evaluation pathway for access to unapproved vaccines [14]. Despite such efforts, as well as support from the International Coalition of Medicines Regulatory Authorities (ICMRA, [15]) and other non-governmental organizations including BMGF, GAVI, IAVI, IVI, COVAX, and CEPI, the COVID-19 pandemic highlighted the tenuous nature of regulatory oversight. Prior to the next influenza pandemic, it is critical to ensure that, where needed, all countries are supported both to strengthen their regulatory frameworks and capacity (particularly for GMP inspections), and to streamline the review, emergency access, and approval processes needed for pandemic vaccines. 

Enhanced global regulatory capacity and collaboration will be key to accelerating equitable access to pandemic vaccines. WHO has supported both a network of NRAs involved in regulating influenza vaccines and efforts to harmonize regulatory approaches. However, it is the NRAs, not WHO, that are responsible for each nation’s regulatory decisions, and even though NRAs share broad goals, each typically has divergent standards and requirements for emergency authorization and licensing. As a result, manufacturers frequently have to customize their vaccine development and/or their regulatory submissions to meet differing requirements among countries where they intend to distribute their product, causing inefficiencies, duplication, and delays. To evaluate and equitably deliver vaccines more expeditiously for a pandemic, there is an urgent need to develop a global regulatory framework for pandemic influenza vaccines that incorporates the specificity needed to prevent such inefficiencies. Such a global framework must be agreed upon by NRAs before a pandemic to ensure that consistent recommendations and requirements are communicated in a timely manner to developers, guiding vaccine development both prior to and during the pandemic. 

Such an effort can build upon what has already been accomplished by the WHO and NRAs. As a basic requirement, mechanisms for regulatory collaboration, including information sharing, are needed. Agreements among NRAs should be implemented pre-pandemic to enable joint reviews and workload sharing for assessment of preclinical, chemistry, manufacturing and control (CMC), and clinical data, as well as of manufacturing facilities. While final regulatory decisions must ultimately be made at the national level, the scientific reviews themselves could be performed collaboratively by an established international core of regulatory experts. The reviews would ideally utilize a single regulatory dossier, which could be based on the ICH Common Technical Document (CTD, [16]) so that sponsors could submit worldwide to meet requirements of all participating regulatory authorities. This would require advance consensus among NRAs and, preferably, development of common global guidance on the core data and other requirements needed to support emergency authorization and/or licensure of vaccines. Such a process could be coordinated by WHO and/or ICMRA for pandemic influenza vaccines and would require sustainable funding. While there are many challenges, it is essential that the global regulatory community implement creative options for working together, such as those discussed, to optimize pandemic influenza vaccine development and evaluation and the use of limited regulatory resources and expertise.

## 4. Protecting the Integrity of Regulatory Agencies and Processes from External Interference

Regulatory agencies are uniquely tasked with objectively evaluating product safety and efficacy and protecting the public from harmful or ineffective products. This role is critical during public health emergencies, when other stakeholders typically may have conflicting political or economic interests. The COVID-19 experience showed how, in turbulent times, medical products such as vaccines can themselves become politicized or subsumed in other debates, such as over personal autonomy and governmental authority. It is therefore not surprising that regulatory agencies found themselves under intense external pressure from within and outside of governments [17,18,19]. While all regulatory decisions inherently are benefit-to-risk judgements, ones which in an emergency may well need to be made using more limited data and with greater than normal uncertainty, they are intended to be based on an unbiased, data-based, deliberative process, and undermining or threatening that process can erode public trust and confidence, as occurred with COVID-19 vaccines [20].

Additional steps are needed to reduce external pressures on and potential disruption of regulatory decision-making, particularly during emergencies. We suggest careful exploration, through a detailed independent review including outside experts, of threats to regulatory integrity during the COVID-19 pandemic and steps, beyond basic avoidance of conflicts of interest [21], that could be taken to protect this integrity in future emergencies. Examples of potential enhancements that could be considered specifically for the FDA include: (1) Requiring public advisory committee discussions and votes for significant authorizations or approvals related to a public health emergency; (2) Requiring public access to the documentary evidence and basis for decision-making for EUAs and other major decisions; (3) Reducing susceptibility of the FDA Commissioner to political pressure by making the position a term appointment extending beyond one Presidential cycle and/or removing the requirement for Senate confirmation; (4) Providing no fault mechanisms, perhaps analogous to Federal Aviation Administration near-miss reporting [22], for government staff and scientists to report undue political or other pressure to an independent ombudsman or oversight body; (5) Ensuring consequences for violations of policies intended to protect whistleblowers.

## 5. Opportunities for Improvement: Manufacturing Quality, Readiness and Capacity

Vaccines are defined by their manufacturing processes because changes at almost any stage—such as to antigens or other active ingredients (e.g., nucleic acids, vectors), purification, production scale, in-process testing, or final product filling—may lead to unanticipated differences in efficacy or safety. 

Therefore, to reliably produce hundreds of millions of doses that perform consistently, manufacturers must develop, control, validate, and monitor their processes from end to end (Table 2). Process validation is an important element of ensuring control across all manufacturing sites and must be done at scales relevant to those ultimately employed for producing vaccines for population use. By necessity, validation at scale is one of the later steps in process development and can, in cases where clinical development has been accelerated, become challenging and rate-limiting, impacting regulatory review and approval. Ideally, at least the early steps in process validation (including analytical and potency assays) would be completed prior to a pandemic. ICH Q9 provides for risk-based approaches to validation [23]. However, different NRAs have implemented varying requirements for data required and the timing of its availability. To facilitate faster and more equitable vaccine access, it would be beneficial to develop consistent risk-based approaches to process validation that are adopted across all NRAs. 

The production of vaccines must meet requirements of Good Manufacturing Practice (GMP), as defined by regulatory bodies [24]. If not addressed before a pandemic, seemingly minor GMP issues may result in substantial delays and such risks are likely to be higher in circumstances where development is accelerated. For example, determining potency for protein-based antigens proved challenging for several COVID-19 vaccines, affecting both clinical trial results [25] and regulatory assessments [26]. In the US, the IND stage allows the FDA to review not only clinical protocols but also CMC data, starting prior to clinical studies and continuing throughout process and product development. It will be essential for regulatory authorities to review and evaluate CMC for novel vaccine technology platforms, as well as manufacturing process development and facilities (including potential scale-up) to the greatest extent possible during the pre-pandemic period. 

A certain degree of flexibility was applied to COVID-19 vaccines regarding manufacturing process performance and validation, including allowing reference to data from similar products made utilizing the same technologies. This was consistent with guidance that COVID-19 vaccine development could be accelerated based on data from similar products manufactured using well-characterized platform technologies, to the extent scientifically appropriate, reducing the need for product-specific data [7].

Complete validation of manufacturing processes and control test methods is not required by regulatory authorities prior to initial clinical studies. However, even for early clinical studies it must be shown that the product is adequately characterized and is manufactured using well-defined and consistent processes. Similarly, neither final nor complete characterization and specifications of active ingredient(s) are required to initiate clinical studies. The specifications of both active ingredient(s) and final product must be provided and justified prior to authorization or approval, but preliminary acceptance criteria may support an EUA and then be adjusted and narrowed during further development prior to licensure. For example, most NRAs do not expect that final stability and shelf-life studies for the active ingredient(s) and final product will be available prior to Phase 2/3 clinical studies or emergency use. Instead, interim data may support determination of initial shelf life and be updated as additional data become available. Manufacturers should engage early with regulatory authorities to discuss the type and extent of CMC information needed both during development and for potential emergency authorization and licensure.

The FDA granted Fast Track designation for several investigational COVID-19 vaccines. This designation allowed sponsors to submit data (including on CMC) for “rolling review” as it became available and provided early and frequent communication between FDA and sponsors throughout the development process. Such flexible approaches—including for CMC data, which may need to be rapidly developed and then frequently adjusted for vaccine manufacturing and scale up—will be key in enabling a rapid and efficient pandemic response.

Global manufacturing capacity is insufficient and continues to be a largely unaddressed rate-limiting step for pandemic response. The groundwork for enhanced rapid scale-up capabilities—including on-site manufacturing facility pre-inspection (or, at minimum, inspectional readiness) and regulatory confirmation of process validation—should be accomplished during the pre-pandemic period. Collaborative inspections, global information sharing, and mutual recognition of inspectional findings among WHO-designated functional NRAs should be strengthened to support more efficient vaccine evaluation, regulatory decision-making, and availability. Batch release, including specific policies, processes, and criteria, is another critical issue to be addressed ahead of a pandemic. In the case of SARS-CoV-2, WHO proposed emergency measures so that National Control Laboratories could reduce risks of delays in batch-release that may result in product expiry and/or exacerbate shortages [27]. Finally, it is inefficient and confusing for every country to have different product labeling, packaging, and package inserts, and inhibits fungibility of vaccine supplies across borders. It should be possible, as proposed for harmonizing authorization and approval requirements, for interested regulators to collaborate to create a universal label, whether virtual/digital, physical or both, with identical language and content and, where needed, accurate translation. Digital formats could also enable virtual updating of information such as expiry dates and safety data. While such an innovation may require changes in some nations’ regulatory and legal provisions, the benefits, particularly in emergency situations, would be substantial.

Launching a manufacturing facility or site, particularly for production of novel vaccines, is complex and challenging and often associated with major delays. To avoid start up issues, the manufacturing base and surge capacity planned for use in a pandemic should be kept “warm” through active operation prior to a pandemic. Ongoing interpandemic manufacturing of similar vaccine(s), such as seasonal influenza or other vaccine(s) made with the same platform and similar methods, is essential to ensure successful manufacturing when a pandemic occurs. Additionally, there should be contingency planning for provision or stockpiling of other vaccines or biologics which may be displaced if surge manufacturing requires use of capacity borrowed from routinely produced products. Such warm base and contingency planning should also be in place for related manufacturing processes (e.g., vial fill and finish), materials (e.g., media, disposables), and supplies (e.g., vials, stoppers, syringes, etc.) and include the capacity needed to support increased manufacturing at the anticipated massive global scale. 

During the COVID-19 pandemic, unprecedented demand and pandemic related disruptions of workforces and transportation resulted in numerous local and global supply chain issues. For example, disposable cell culture components, culture media, capacity for filling, finishing, and labeling of products were each limiting in some COVID-19 vaccine scale-up efforts. A complete analysis should be done of planned manufacturing processes from end to end, with the engagement of regulators. Unique solutions should be considered. For example, CEPI worked to organize an anonymized, confidential global exchange system for vaccine production ingredients and components [23], and the US government supported excess fill and finish capacity that could be enlisted by those in need [24]. Similarly, governments, public health agencies, manufacturers and regulators should work together to evaluate opportunities for utilization of both equipment (e.g., disposable vessels, tubing, and related commodities) and ingredients that are potentially interchangeable rather than specific to a particular product, process, facility, or piece of equipment. Approaches such as development of global standards for such equipment, or in-advance testing and evaluation to support regulatory flexibility in emergency situations, should be considered.

## 6. Opportunities for Improvement: Preclinical Studies

Once a vaccine’s manufacturing process is sufficiently characterized (i.e., comparable to planned clinical use material with respect to physicochemical characterization, stability, and formulation), preclinical testing (including in animal models) is typically performed to define the vaccine candidate’s initial safety and immunogenicity profile (Table 3). Preclinical studies can help identify potential safety risks as well as guide dose selection and regimens to be evaluated in clinical studies. For example, studies in some animal models of vaccine candidates against coronaviruses such as SARS-CoV and MERS-CoV raised safety concerns related to vaccine-associated enhanced respiratory disease (ERD) [28]. As a result, FDA and other regulators requested animal studies to characterize the vaccine-induced immune response of COVID-19 vaccine candidates with respect to immune markers potentially predictive of ERD, such as total vs. functional (e.g., neutralizing) antibody and Th1/Th2 T cell balance [7]. Preclinical studies may also utilize unique model systems, such as those designed to include relevant viral receptors or humanized immune response components, with the intent of contributing to understanding both the disease and host immune responses likely to predict clinical benefit. Preclinical testing may identify specific safety concerns that inform the design of human clinical studies and evaluate potentially deleterious immune responses, as reported for a formalin-inactivated RSV vaccine candidate which elicited Th-2 hypersensitivity [28]. 

The extent of preclinical data required prior to first-in-human (FIH) clinical trials depends upon the vaccine construct and the supportive data available for it and closely related vaccines and/or pathogens. For a novel vaccine candidate for which no prior preclinical or clinical data are available, animal safety studies would normally be required prior to FIH trials. In other cases, it may not be necessary to perform such studies prior to FIH trials because adequate relevant information on safety may be available from other sources. For example, if a vaccine candidate is made with a platform technology already utilized to manufacture either a licensed or well-studied investigational vaccines, it may be possible to use preclinical data (e.g., from toxicity and biodistribution studies) as well as clinical data using the same platform to support FIH clinical trials for the new candidate. 

The potential use, and therefore safety, of vaccines in women of childbearing potential and during pregnancy was an important challenge in the COVID-19 response [29] and will be just as important during an influenza pandemic. Fortunately, current seasonal influenza vaccines have accumulated extensive relevant data and experience in pregnancy. As a result, animal developmental and reproductive toxicity (DART) studies are unlikely to be required for pandemic influenza vaccines made based on these technologies. However, for newer vaccines and technologies DART studies are likely to be required prior to studies and use in pregnant humans. Such studies should, if possible, be performed prior to the pandemic, and could significantly speed early access to vaccines for this population, which typically has an elevated risk of severe outcomes from influenza infection. Similarly, biodistribution studies in animals may be needed and should be performed pre-pandemic if a vaccine construct is novel and there are insufficient data from other uses of the platform technology. 

The predictive value of animal studies of influenza vaccines may be impacted by the fact that, as opposed to most laboratory animals, which are immunologically naïve, most humans will have been previously exposed to influenza viruses and have pre-existing immune memory, even if not for the pandemic strain, which may influence the response to vaccination [30]. As noted, a general principle for preclinical studies should be to perform them, wherever feasible, prior to the next pandemic. In addition, studies should be performed as consistently as possible across different products. This can be facilitated by governmental funding and coordination, including pre-competitive access to shared viral strains, antigens, immunological assays, reagents and reference sera, animal model protocols, and resources.

## 7. Opportunities for Improvement: Clinical Development 

Routine vaccine clinical development has historically been a multi-step, multi-year process that, from start to finish, includes protocol design, site start-up, institutional, IRB and regulatory approvals, and recruitment, enrolling, and following subjects for the time needed to meet safety and efficacy endpoints, with curation and analysis of data. In contrast, for vaccines developed in response to an outbreak or pandemic, some clinical data typically available prior to vaccine use in more routine vaccine development programs may not be initially available when the vaccine is first used. For pandemic vaccines, the priority will be to ensure that key efficacy and safety requirements have been met. Other potentially important information such as on duration of protection, optimal dosing schedules, the necessity for boosters and use in some special populations, may only become available as products are utilized. 

While the development of COVID-19 vaccines was, compared to routine development, highly accelerated (e.g., for mRNA vaccines from pathogen sequence to start of clinical trials in as little as two months, and to first broad population use under EUA in less than a year), clinical studies were still the most time-consuming part of initial development. Multiple opportunities exist to enhance the speed and quality of pandemic influenza vaccine clinical development (Table 4).

The major challenge is the time required to achieve the fundamental goals of documenting safety and efficacy, with the latter based either on inference from likely immune correlates of protection or on actual clinical endpoints such as infection and disease. Despite unprecedented collaboration, intensive regulatory interactions, and massive resources, sufficient documentation of safety and efficacy of US-authorized COVID-19 vaccines still took 7–9 months from first dose in humans to issuance of EUAs. However, in the H1N1 pandemic of 2009, Phase 3 safety and efficacy trials were, as discussed, not required for vaccines manufactured using the same process as licensed HA-based seasonal influenza vaccines. This allowed clinical development to be shortened to approximately 2 months from the start of the needed Phase 1–2 trials establishing dosing and immunogenicity to regulatory approval of the pandemic vaccines as a “strain change”. This pandemic strain-change pathway remains viable for licensed HA-based vaccines. 

For currently unapproved influenza vaccines that may be more effective and/or scalable, one of the highest priority strategies should be to complete as much clinical development as possible during the pre-pandemic period. Ideally, as for HA-based vaccines in 2009, any such vaccines using novel technologies and/or alternative immune targets, including their associated manufacturing processes and facilities, should be studied, and licensed for seasonal use (or as dedicated pre-pandemic vaccines) prior to the pandemic. Licensure of potentially improved vaccines for seasonal use, and broader population uptake in the US and globally, would also be extremely beneficial in supporting a warm manufacturing base. The data to support such approvals should include large safety databases (e.g., at least several thousand, as required for COVID-19 vaccines) and documentation of clinical efficacy. In addition, it is highly desirable that likely surrogate markers of protection be identified that could then expedite EUA or approval for a vaccine based on an emerging pandemic variant, similar to a strain change for annual influenza vaccines. Identification of surrogate markers can also expedite authorization or approval of additional vaccines using the same technologies. The COVID-19 experience also illustrated how important it is to obtain pre-pandemic data to support vaccine use in special populations such as children, elderly, pregnant, and immunocompromised individuals, and to potentially differentiate vaccines that may be advantageous for specific populations.

Even if a pandemic influenza vaccine is made using the same processes as a licensed seasonal one, it may not perform identically. Unique antigens to which there has been little prior population exposure may be poorly immunogenic and/or require higher or multiple doses, as seen with H5N1 avian influenza [31]. Different viruses, antigens or, conceivably, genetic sequences may present unanticipated manufacturing challenges. In addition, important standards and assays, such as those used during manufacturing or to measure vaccine potency or clinical immune responses, may need to be strain-specific and can be time consuming to develop, validate and produce. Thus, whenever possible, such standards and assays should be developed pre-pandemic and made freely available, including to allow comparability across multiple vaccines (as currently done for seasonal influenza vaccines through WHO Collaborating Centers). In addition, some of the same reagents, assays, and samples used in vaccine development (e.g., antibody assays, clinical samples) may be important in the development of therapeutics and diagnostics. Thus, there should be coordination across regulatory and scientific domains such as the FDA, CDC, and NIH, as well as with global regulators and organizations such as the WHO and CEPI. 

Pandemic influenza vaccines could become available much earlier in a pandemic if support is provided in the interpandemic period to complete Phase 1–2 dosing and immunogenicity studies of prototype vaccines manufactured based on novel influenza variants of pandemic concern. Such studies could include a complete library of known potential virus types and/or prioritize those identified by the WHO and CDC as of particular concern [32]. 

When a pandemic strikes, there may be promising vaccines in early development stages and/or that are not being developed commercially for seasonal influenza. Such vaccines could, for example, include those based on novel but unproven approaches that might improve efficacy and/or achieve protection against a broader array of HA types. They might possess manufacturing, thermal stability, dosing regimen, or administration route attributes of particular value for pandemic use. Previously unlicensed mRNA and viral-vectored vaccines against SARS-CoV-2 presented a similar challenge at the start of the COVID-19 pandemic, and insufficient prior investment in clinical development and validated manufacturing processes contributed to the approximate one-year gap from pathogen identification to vaccine authorization. A number of mRNA-based vaccines are currently being commercially developed for seasonal influenza and, if successful, could provide the needed groundwork to speed development of and access to pandemic influenza vaccines. While their success could be transformative, it is by no means assured.

Development of some technologies or vaccines, especially if specifically for pandemic influenza, and if not commercially driven by a goal of marketing for seasonal influenza, will likely need to be incentivized by or performed in partnership with governments and/or NGOs. For such vaccines to be rapidly deployable in a pandemic, studies relevant to future pandemic use (e.g., including of candidate pandemic strains) should also be performed before the pandemic and include, at minimum, proof of principle in animal models followed by Phase 1–2 human studies to define dosing and immunogenicity. If Phase 3 efficacy studies have not been performed against seasonal influenza, it would still be helpful and informative, and support future EUA, to build a substantial safety database on the vaccine or, at minimum, the platform. 

With respect to efficacy, human challenge studies, perhaps utilizing seasonal influenza viruses, may also play a role, at least perhaps in helping identify or reject approaches that do not achieve protection at least similar to available approved vaccines. However, pre-pandemic use of pandemic threat viruses in challenge studies raises serious biosafety issues and may not be feasible. In addition, available challenge models may, like animal studies, differ in ways (i.e., inoculum, route, and disease pathogenesis) that make clinical efficacy against natural infection difficult to reliably predict. 

Several opportunities exist to improve the speed and efficiency of clinical development, whether pre-pandemic or for those studies that still may be needed during a pandemic. The NIH’s use of existing networks to support COVID-19 vaccine trials is a good example. Further, entities such as WHO, through its Solidarity trials [33], and the United Kingdom, through its Recovery network [34], were able to rapidly start up and perform trials employing novel (e.g., adaptive, multi-arm) as well as simplified and relatively inexpensive study designs. While there were some such successes in the US, including use of relatively seamless multi-phase studies, in other cases important clinical questions (e.g., mixing and matching of different vaccines, studies of dosing schedules not in manufacturers’ protocols) were not prioritized or answered in a timely manner and to date have been addressed only through small studies providing limited data for decision-making.

The US should support increased and more agile domestic capacity to perform rapid studies of pandemic vaccines (as well as therapeutics, diagnostics, and clinical interventions) and would benefit from further engagement and collaboration with international efforts. Both US and globally integrated capacity should, where feasible, build on existing capabilities and be structured to enable both emergent studies during a pandemic and other research during interpandemic periods, i.e., as a warm base for clinical trials. Particularly for Phase 3 pivotal trials, rapid enrollment, and ability to direct studies across the globe to areas with ongoing or predicted disease outbreaks could greatly accelerate time to actionable trial results. In addition, globally distributed trials can foster inclusion of diverse and representative subjects and meet regulatory requirements to bring a product to market in new areas. However, it is critical to understand the global regulatory environment, and, where feasible, seek consistency across trials and geographies, so that needed products may be developed efficiently, become widely accessible within similar time windows, and be used with global confidence. In addition, it should be ensured that pivotal trials are conducted to meet standards for good clinical practices and to address ethical requirements, wherever they are conducted.

Further, these efforts, where feasible, should engage large health systems and electronic health record holders (e.g., in the US those such as the Veterans’ Administration (VA), Cerner and Epic) and involve communities and participants from diverse populations. Such improvements—including to help extend the reach of trials beyond predominantly academic, urban-based capabilities—are urgently needed and can, as noted, be configured to serve a dual purpose with funding and operational provisions that support interpandemic use but allow them to be rapidly redirected in a pandemic or other public health emergency. For studies that cannot be performed in the pre-pandemic period (e.g., Phase 1–2 trials of vaccines against a wholly unanticipated pandemic strain), study sites, protocols, implementation plans, and IRB approvals can and still should be in place and ready prior to the pandemic. 

Great care should be put into developing clinical protocols incorporating scientific, regulatory, and public health input, and addressing issues such as the endpoints to be measured and immune and other assays employed. Such up-front investment will enhance the utility of study results for regulatory and public health decision-making and their comparability across vaccines and populations. The potential of such approaches was shown in NIH-supported COVID-19 vaccine trials. However, some important studies both in the US and globally utilized different disease endpoints and definitions as well as differing immune assays, making comparability and interpretation difficult. Government and NGO investments such as those made through OWS or CEPI, as well as overriding public health and national security interests, should be harnessed to provide motivation and leverage to better ensure that study design and governance are optimized to achieve public health goals. 

Furthermore, consistency and shared governance across trials can empower efficiencies such as use of common control populations and adaptive designs, including to move new products into studies as they become ready and to rotate out products appearing futile or inferior. In addition to adaptive designs, serious consideration should be given to the potential of “large simple trials” [35] for pandemic vaccines. Compared to traditional Phase 3 studies, large simple trials may be more feasible, faster, less expensive, and more valuable in assessing efficacy and important safety issues. It may be preferable, for example, to perform a very simple 100,000 subject trial that enrolls patients and measures selected major safety and efficacy outcomes using electronic health records, such as may be achievable through the VA and other large health systems, than a more in-depth typical Phase 3 trial of 10–20,000 subjects. This approach may be particularly useful for previously unlicensed vaccines and technologies where limited pre-pandemic data are available as its power to define efficacy and to detect and quantify relatively rare vaccine related adverse events (such as the myocarditis or thrombosis seen with some COIV-19 vaccines) is significantly enhanced. Such approaches merit careful consideration and would benefit from regulatory and governmental support and advanced engagement and planning, including for needed resources, with health care organizations. Otherwise, sponsors and investigators tend to do what is perceived as providing the least regulatory and scientific risk. 

It is not reasonable to expect industry or academic sponsors or investigators to identify cross-cutting public health needs and to prioritize, invest in, or pursue certain types of studies (e.g., mixing and matching of different vaccines, alternative immunization schedules, and comparison of outcomes and duration of protection among different vaccines). Therefore, engagement of governments and public health in defining such research questions as well as needed trial design and conduct is critical. Such engagement must address industry and investigator concerns and be managed so as to not slow initiation of trials, rather than as business as usual. Mutually acceptable governance and approaches to operational matters (e.g., common protocols and definitions, clinical data reporting and sharing with US agencies, and coordination of communications) can, with suitable incentives, be built into contracts and/or advanced purchase commitments. 

One specific unanticipated challenge that arose from the early success of COVID-19 vaccine Phase 3 trials was the desire to cross placebo recipients over to active immunization, unblinding them after only approximately 2 months of follow-up. This issue should be addressed in future design of RCTs, and consideration given to potential approaches to reduce impacts of unblinding, such as cross over of all patients (e.g., also crossing vaccine patients over to receive placebo). This type of issue, and the abbreviated follow-up of RCT participants, also highlight the importance of post-authorization or post-approval, “real-world” safety, and effectiveness data to evaluate longer-term outcomes and issues such as the durability of protection over time and against emerging variants. Again, this need goes beyond the incentivization or abilities of individual manufacturers and calls for common approaches and investments driven by public health priorities.

## 8. Opportunities for Improvement: Post-Authorization/Approval; Data and Monitoring in the Pandemic

The COVID-19 experience amply illustrated that monitoring the safety and effectiveness of vaccines during and after their initial rollout is critical to inform regulatory and public health decisions and to maintain public confidence. It would be preferred that any pandemic influenza vaccine be well studied prior to the pandemic. At one extreme, a licensed vaccine based on widely used technologies should have a well-defined safety profile from use in millions of individuals over many years. As in the 2009 H1N1 influenza pandemic, this should *prima facie* provide a high degree of confidence, particularly with respect to safety. However, some people will remain concerned that pandemic vaccines have been rushed and seem new or experimental. Furthermore, even a well-characterized vaccine will utilize an antigen or genetic sequence based on a pandemic strain that has likely not been widely used, and in a unique time and setting. Therefore, unexpected vaccine-related adverse events (AEs) may still occur, as was seen with excess vaccine associated Guillain-Barre syndrome in the 1976 Swine influenza outbreak [36] and with narcolepsy associated with one adjuvanted influenza vaccine in the 2009 influenza pandemic [5]. In addition, the effectiveness of current influenza vaccines varies depending on factors such as the viral antigen and the age and immune status of recipients and, therefore, will be initially uncertain and require monitoring during a pandemic. At the other extreme, vaccines initially used during a pandemic may, as was the case with COVID-19, be based on novel technologies, with far more limited data and experience, and made available through emergency use provisions, making comprehensive and near real-time monitoring of safety and effectiveness, including duration of protection, even more important. 

In the case of COVID-19 vaccines, because they were previously unlicensed and utilized technologies without an extensive track record, the FDA required large, well-controlled trials, including short-term safety and efficacy data, even for EUA [8,9]. Although the trials included safety databases large enough (e.g., 10–20,000 vaccinated individuals) to identify common adverse events, they did not have the size or scope to detect or define the nature or rates of rarer but potentially serious vaccine-related AEs, including events that might occur primarily in certain subpopulations. Post-approval or EUA safety monitoring systems not only need to be able to promptly detect such events, including those of an unanticipated nature, but also must provide data to allow authorities to evaluate and respond to events that may not be caused by vaccine but be coincidental or even non-factual, including rumors and misinformation appearing on social media [37]. For all these reasons, robust and near real-time safety monitoring and analysis are critical, particularly as vaccines are rolled out to the first several hundred thousand to millions of recipients, as is transparent, trusted, and effective communication of the findings. 

To be effective, particularly early in an accelerating vaccination campaign, such monitoring will require that data are captured from much of the vaccinated population. This is beyond the capabilities of manufacturers, who typically support more limited post-approval studies. Furthermore, while analysis of passively collected adverse event reports, such as through the Vaccine Adverse Event Reporting System (VAERS), can be important in signal detection, it usually cannot assess causation given the inherent shortcomings of spontaneous reporting. Nor can passive reporting provide the precision to detect potentially significant changes in the incidence of common health events. Therefore, active monitoring using systems which capture data from large numbers of individuals, and allow ascertainment of their demographics, vaccination history, and the specific adverse events, are a critical component of safety monitoring. National health and informatics systems help make it more feasible for some countries, such as the UK, to actively monitor vaccine safety and effectiveness. However, In the US, with its more fragmented health care and informatics, such monitoring is more challenging. Data to support active monitoring in the US can be acquired in two main ways: using claims information from payors or insurers, such as Medicare data from the Centers for Medicare and Medicaid Services (CMS), and/or using information from electronic health records (EHRs). Claims databases typically have an advantage of very large numbers but provide less granular data than EHRs. Different databases also may have important differences in demographics (e.g., age, race, ethnicity, underlying conditions) and thus, when planning for monitoring, data should be acquired in a manner to obtain a complete picture and, in fact, may be selected to enrich for subpopulations of special interest (e.g., pregnancy, specific age groups, etc.). Active vaccine safety monitoring was pioneered through CDC’s longstanding Vaccine Safety Datalink (VSD) system which uses EHR based data [38], through FDA’s partnership with the CMS using claims data [39], and through the FDA-supported PRISM system, using both data types [40]. 

Principles for employing such resources in a public health emergency were put in place for the 2009 H1N1 pandemic and included both passive surveillance through VAERS and active surveillance through CMS claims data and EHRs from managed care organizations, including VSD. A governance and communications framework was put in place within the Department of Health and Human Services (HHS) Secretary’s National Vaccine Program Office (NVPO). NVPO coordinated an interagency group of experts that regularly analyzed results and reported to independent advisors and the public through the National Vaccine Advisory Committee [41]. This system monitored and communicated about vaccine safety in near real time and was able to inform and reassure the government and public as to the safety of the vaccines. 

During the COVID-19 pandemic, similar approaches were used and broadened to include larger numbers of vaccine recipients. In general, these approaches worked well. However, such efforts were not supported by OWS or other dedicated funding, were largely left to individual federal agencies, and lacked an overall strategic approach. They started slowly, with insufficient subjects monitored and breadth of data capture, only fully ramping up well after vaccines became available. Some data-streams, such as from the CMS and from FDA’s updated claims and EHR-based systems (BEST) were delayed for unclear reasons. This meant that early safety investigations largely relied on passive reporting from VAERS and, for active surveillance, on VSD, which still covers a relatively limited population (approximately 12 million or 3.6% of the US population). Not surprisingly, some relatively rare AEs only became evident, and could only be well characterized as to their incidence, following months of widespread use of specific COVID-19 vaccines under EUA. To date, these AEs have included myocarditis following mRNA vaccines, particularly affecting young males after a second dose [42]. Also noted have been thrombosis with thrombocytopenia, particularly affecting young women [43]; and Guillain-Barre syndrome [44], both following use of adenovirus-vectored vaccines and initially brought to attention through non-US based vaccine use and reporting. In support of vaccine safety efforts, the Brighton Collaborative worked with CEPI to establish common definitions for potential AEs [45] and efforts were made to better understand background rates of potential AEs in the unvaccinated population [46]. However, to improve AE detection and analysis and ensure comparability across populations and vaccines, AE definitions for potential events of concern should, to the greatest extent possible, be agreed upon and in place before the pandemic. In addition, contemporary background rates, rather than historical sources, should be available from the same populations and datasets that will be used to track AEs in the pandemic. In addition, monitoring capabilities and plans should include both knowing what data will be monitored and prospective analytic plans with pre-specified times or subject numbers to trigger routine analyses. 

As illustrated by the COVID-19 vaccine experience, access to more data and enhanced analytics can improve the detection, investigation, and analysis of pandemic influenza vaccine-related AEs, and in turn the speed and quality of regulatory assessments and decision making, as well as immunization recommendations and related communications. This can be accomplished through earlier and more substantial investment in both data access and analytic methodologies. This effort should further engage EHR systems and data holders not involved to date, an effort which, like VSD, is well worth supporting before the next pandemic. While current methodologies are powerful, tools like artificial intelligence and machine learning (AI/ML) may further enhance the utility of large data sets and merit continued exploration. All such investments in data and analytics are “dual use” as they can help better monitor vaccine and other medical product safety in interpandemic periods. 

In general, the datasets and approaches needed to better empower safety monitoring can also improve the monitoring of vaccine effectiveness (below). Therefore, a national strategy, along with funding and implementation plans, should be developed in collaboration across government and private health, payor, and health IT sectors to ensure access to such data and the methodologies to analyze it. Such investments would pay off both in safety and in public trust. Furthermore, as noted, unique data and resources are available in other countries and regions, which may roll out different vaccines at different times and detect and define safety issues prior to the US. The US should strategically collaborate with international partners to ensure detection of, and communications about, safety issues are optimized and based on truly global situational awareness. Again, just as a ‘warm base’ is needed to support manufacturing and clinical trials, a global warm base for needed data and analytics is essential to monitor vaccine safety and effectiveness during public health emergencies. 

Beyond enhanced data collection and analytics, it is also worth reflecting on how vaccine safety efforts are governed and communicated. For the COVID-19 outbreak, similar to the 2009 influenza pandemic, an expert technical advisory group with nongovernmental members and government liaisons was brought together to evaluate safety data [47]. A difference between the COVID-19 pandemic and the approach to the 2009 influenza pandemic was that the COVID-19 interagency safety group was under the auspices of the CDC, rather than the HHS Secretary, and reported through the CDC ACIP, the same advisory committee making immunization recommendations. While this was managed well and engaged appropriate experts, the governance and location of such efforts may benefit from re-elevation to the HHS Secretary level. This can help ensure all agencies are heard and any differences adjudicated. Such governance may also help provide an opportunity for high-level ownership and accountability in meeting resource needs, which were largely ignored early in the COVID-19 pandemic. In addition, high-level coordination can help ensure consistent whole-of-government communications, including with international public health partners. Finally, such an approach could enhance the perception of the priority and integrity accorded to safety.

The COVID-19 pandemic also graphically illustrated the importance of being prepared to monitor vaccine effectiveness (VE) following vaccine roll out. Initially very high estimates of VE were based on short-term data and interpreted overconfidently. In addition, while studies deliberately included elderly subjects, some other subpopulations at high risk of severe disease, such as those with multiple co-morbidities and the highly immune compromised, were underrepresented or excluded. Furthermore, for the newly authorized technologies, there was no track record concerning the duration of vaccine protection, nor could the trials predict efficacy against unanticipated emerging viral variants. Unfortunately, as the world faced both the emergence of new variants and the apparent waning of vaccine-induced protection, the US strategy and capability to track VE was limited. Initially, VE monitoring primarily utilized a few sites that monitored seasonal flu vaccine efficacy which covered relatively small populations and provided insufficient capability for rapid, generalizable analyses. Thus, the detection of waning VE first came largely from other parts of the world [48], and the US could not initially determine the degree to which VE might be waning domestically. Initially, the limited US data were available primarily from specific localities or health systems, sometimes with conflicting results. Fortunately, additional capabilities were brought to bear (including by CDC and through both networks of health systems and states), ultimately improving US situational awareness. However, limitations in the strategy, timeliness, and scope of VE monitoring contributed to confusion and delays in regulatory and public health decision-making and communication, for example, concerning “booster” doses for mRNA vaccines [49]. 

Observed limitations in VE monitoring and potential improvements have many commonalities with those discussed for safety. Foremost, there is a need for a clear strategy for VE monitoring of future pandemic influenza vaccines. To the extent VE data sources and analytics overlap with safety data needs, investments in data access and monitoring plans can and should be integrated. Such a strategy should drive enhanced access to and analysis plans for relevant data, with sources ranging from federal data, states, health care systems with EHRs, and potentially, EHR providers. Major challenges exist, such as the ability to cross-reference individuals’ immunization histories (including product identification) to testing and health outcomes. These challenges are daunting in a fragmented care system, but the COVID-19 experience illustrates it can be done. 

While highlighting the need for enhanced capabilities, the COVID-19 VE monitoring experience also revealed methodologic challenges that, where possible, should be addressed ahead of the next influenza pandemic. As already noted with respect to both designing clinical trials and tracking safety, consistent definitions and clear endpoints are critical. Indeed, confusion has been widespread as to the desired goals and outcomes of immunization and has directly carried over to VE monitoring. That is, were vaccines expected to prevent all infections and to reduce transmission, or were vaccines to primarily protect against more severe outcomes (e.g., hospitalization and death) and health system stress? Both types of outcomes can and should be monitored to provide a complete picture, but have different implications for data needs and, importantly, when communicating with the public. 

Other significant methodologic challenges remain. VE studies inherently compare infection or disease-related outcome rates in vaccinated and unvaccinated individuals (or compare vaccination rates in infected vs. uninfected individuals). While randomized controlled trials (RCTs) greatly reduce the risk of vaccinated and unvaccinated individuals differing in ways other than being vaccinated or not, observational studies used to measure VE introduce multiple factors that may confound exposure and potential differences in infection and disease risk, causing major challenges in interpreting results. Various approaches can be used to help correct for such differences [50] and include matching of vaccinated and non-vaccinated controls for a variety of factors, multi-variate and sensitivity analyses, propensity scoring, and use of test negative study designs that compare vaccination rates among otherwise matched individuals with and without the infection/outcome of interest. These methods reduce but cannot eliminate confounding. Particularly relevant, as seen during COVID-19, is that individuals’ vaccination decisions may become embroiled in a complex politically and socially tinged milieu. In such a situation, significant differences between the vaccinated and unvaccinated become increasingly likely, including in behaviors and exposure that may, independently from vaccination status, affect risks of infection and disease as well as the detection and measurement of outcomes (for example, use of testing). Additionally, adding complexity in a pandemic, VE analysis must be performed in a manner that accounts for near constant temporal and dyssynchronous geographic change in infection risk, including changing transmission levels, variants, and population immunity, as well as factors affecting diagnosis such as access to and use of testing. Addressing these issues is methodologically challenging and, as for safety analyses, may benefit from application of AI/ML to the large amounts of relevant data. 

As for vaccine safety, building robust VE monitoring requires a coordinated strategy and implementation plan engaging the full scope of government and health sector stakeholders (Table 5). VE monitoring efforts should also include coordination with global partners, who, as seen during the COVID-19 pandemic, may have unique and/or complementary data that provides enhanced awareness. Both domestically and globally, data sharing and coordinated analysis and communications can be challenging, so relevant agreements should be developed prior to the pandemic. Further, as discussed for safety monitoring, VE monitoring requires a warm base to ensure its utility and rapid start up in a pandemic and can provide dual use capabilities for monitoring other medical products used in the interpandemic periods.

## 9. Conclusions

We have highlighted diverse opportunities, from a regulatory perspective, to accelerate and improve development, evaluation and access to vaccines for the next influenza pandemic. These include the need to ensure agile regulatory policies, standards, and information sharing, globally harmonized to the greatest extent possible, while supporting nations’ regulatory capacity and integrity. We also identify multiple opportunities to accelerate and enhance vaccine pre-clinical and clinical development and manufacturing, as well as the monitoring of safety and effectiveness as pandemic vaccines are rolled out. To be most effective, the needed steps forward should be part of a clear strategy with sufficient long-term resources, be well underway prior to the next pandemic and be globally collaborative and coordinated. A key goal should be, wherever feasible, that pandemic vaccines, even those based on novel and/or unlicensed technologies, have been comprehensively tested during the pre-pandemic period and are ready to be produced at scale using methods and facilities already approved and used for seasonal influenza or other vaccines. However, we must also be prepared to develop and evaluate novel vaccines that may not have been adequately studied before the pandemic, and for unexpected influenza strains that may differ in their clinical manifestations, immunogenicity, or manufacturing. Thus, preparing ahead to the greatest extent possible is essential, while also ensuring capacity and accelerated processes to deal with the unexpected. Taking the steps discussed can facilitate the rapid vaccine production, evaluation, access and monitoring needed to save many lives and protect society in a pandemic, whether due to influenza or another pathogen, while enhancing scientific and public confidence in vaccine safety and effectiveness.

## Figures and Tables

**Table 1:** Selected Opportunities for Strengthening National Regulatory Agencies.

Policies	Capacity, Response, & Collaboration	Integrity
All NRAs should have applicable regulations to facilitate accelerated vaccine development and evaluation to support approval and/or emergency access.	Enhance global NRA capabilities in vaccine review and manufacturing oversight Convene NRAs to develop and promulgate globally agreed guidance on requirements for approval and/or emergency use of pandemic influenza vaccines. Develop a template, such as the CTD, for a dossier that could be submitted to all participating NRAs. Establish agreements to enable confidential information sharing and joint reviews and GMP inspections across NRAs.	Conduct a review of threats to regulatory integrity during COVID-19. Ensure all NRAs have strong conflict of interest protections. Consider enhancements to transparency and integrity, for example: requiring public data access for major decisions during emergencies requiring presentation of data to independent expert advisors protecting agency leaders from political influence ensuring mechanisms for NRA staff to raise concerns about undue influence or coercion without fear of retaliation

**Table 2:** Selected Opportunities to Strengthen Manufacturing Capacity and Regulatory Readiness.

Capacity	Regulatory Readiness
Develop global standards for manufacturing, equipment, and testing Enhance rapid scale-up capabilities Maintain warm-base manufacturing in multiple inspected or inspection-ready locations across the globe Enable global sharing of critical equipment and raw materials Contingency planning and stockpiling for raw materials and supplies and for routine vaccines that may be displaced by pandemic manufacturing Build redundancy and interchangeability in key supply chain components	Harmonized regulatory requirements for acceptable process validation during the pre-pandemic period. Collaborative inspections and global information sharing, e.g., mutual recognition of inspectional findings among WHO-designated NRAs Harmonized international batch release criteria and acceptability, including specific policies, processes, and criteria Harmonized, and where feasible, universal product labeling.

**Table 3:** Selected Opportunities to Improve Preclinical Development and Evaluation.

Pre-Pandemic Preparation	Expedited Regulatory Evaluation
Identification and development of appropriate animal models. Conduct developmental and reproductive toxicity studies, if needed, for novel vaccine platforms. Conduct biodistribution studies for novel constructs, if needed Identify potential immune response safety concerns to inform human clinical studies	Develop global consensus on requirements for preclinical testing of vaccines for pandemic use. Minimize differences in requirements across NRAs for preclinical safety evaluation. Develop common animal study protocols and shared testing reagents Utilize preclinical data from other related products to reduce or eliminate the need for preclinical data for similar products

**Table 4:** Selected Opportunities to Improve Clinical Development.

Conduct Key Studies Pre-Pandemic	Enhance Clinical Study Speed and Quality
Clinical data to support approval, or, if not yet feasible, to support platform: Safety data on several thousand participants Documentation of clinical efficacy (e.g., for seasonal influenza) Studies in special populations, e.g., pregnancy, children Support rapid pathways and readiness to approve vaccines for new strains: Identification of likely surrogates of protection Pre-pandemic Phase 1–2 dose and immunogenicity studies of prototypic pandemic strains Develop and share standardized assays and reagents needed for trials: e.g., potency, immune response measurements Provide global regulatory guidance on clinical data required for approval, emergency use, and strain changes	Support a globally coordinated warm base for large, agile trials: Build on existing capabilities Use in interpandemic for pre-pandemic/other studies with ability to shift to pandemic use Strengthen geographic and population diversity (e.g., rural, non-academic sites, special populations, health systems, EHR holders) Shared governance to identify and prioritize study questions, to optimize study design, endpoints and assays, and to foster comparability across studies Consider and enable novel study approaches e.g., adaptive designs, common controls, large simple trials, agile targeting to outbreak areas

**Table 5:** Selected Opportunities to Improve Safety and Effectiveness Monitoring.

Develop strategic and implementation plans and ensure needed resources for monitoring Increase populations actively monitored to allow capture of data from majority of immunized individuals early in pandemic ○ Include major holders and systems for both EHR and claims data For safety, systems should utilize current or recent control comparison rates in the monitored population rather than historical data Utilize pre-specified, harmonized definitions and data elements for both adverse event and effectiveness endpoints Maintain enhanced monitoring systems as a ready “warm base” used for monitoring safety and effectiveness in interpandemic period Support R&D on artificial intelligence and machine learning approaches to better analyze large data streams and address confounding in observational data Coordinate and collaborate in both information sharing and public health communications with global partners Consider re-elevating US vaccine safety monitoring and communications to the HHS Secretary level
